# Supplementary material for: Iterative improvement in the automatic modular design of robot swarms
Source: PeerJ Comput Sci. 2020 Dec 7;6:e322. doi: 10.7717/peerj-cs.322 (PMC7924708; doi:10.7717/peerj-cs.322)
Supplement: Supplemental Information 3 [file peerj-cs-06-322-s003.zip › argos3/doc/api/standalone/a00514_source.html]

ARGoS: plugins/robots/generic/control\_interface/ci\_range\_and\_bearing\_actuator.cpp Source File


- Main Page
- Related Pages
- Namespaces
- Classes
- Files

- File List
- File Members

# plugins/robots/generic/control\_interface/ci\_range\_and\_bearing\_actuator.cpp

Go to the documentation of this file.

```
00001 
00007 #include "ci_range_and_bearing_actuator.h"
00008 
00009 #ifdef ARGOS_WITH_LUA
00010 #include <argos3/core/wrappers/lua/lua_utility.h>
00011 #endif
00012 
00013 namespace argos {
00014 
00015    /****************************************/
00016    /****************************************/
00017 
00018 #ifdef ARGOS_WITH_LUA
00019    /*
00020     * The stack can have one or two values
00021     * - The case of one value is when you set an entire array
00022     * - In the case of two values, you must have:
00023     *   1. the idx of the data item to set
00024     *   2. the value of the data item in the range [0,255]
00025     */
00026    int LuaRABSetData(lua_State* pt_lua_state) {
00027       if(lua_gettop(pt_lua_state) == 1) {
00028          /* Check parameters */
00029          luaL_checktype(pt_lua_state, 1, LUA_TTABLE);
00030          /* Get reference to actuator */
00031          CCI_RangeAndBearingActuator* pcAct = CLuaUtility::GetDeviceInstance<CCI_RangeAndBearingActuator>(pt_lua_state, "range_and_bearing");
00032          /* Check whether sizes match */
00033          if(pcAct->GetSize() != lua_rawlen(pt_lua_state, -1)) {
00034             return luaL_error(pt_lua_state, "robot.range_and_bearing.set_data(array) expects an array of %d numbers", pcAct->GetSize());
00035          }
00036          /* Fill up a byte array, checking that all elements are numbers */
00037          CByteArray cBuf(pcAct->GetSize());
00038          for(size_t i = 0; i < pcAct->GetSize(); ++i) {
00039             lua_pushnumber(pt_lua_state, i+1);
00040             lua_gettable(pt_lua_state, -2);
00041             if(lua_type(pt_lua_state, -1) == LUA_TNUMBER) {
00042                cBuf[i] = static_cast<UInt8>(lua_tonumber(pt_lua_state, -1));
00043                lua_pop(pt_lua_state, 1);
00044             }
00045             else {
00046                return luaL_error(pt_lua_state, "element #%d of the array is not a number", i+1);
00047             }
00048          }
00049          /* Perform action */
00050          pcAct->SetData(cBuf);
00051          return 0;
00052       }
00053       else if(lua_gettop(pt_lua_state) == 2) {
00054          /* Check parameters */
00055          luaL_checktype(pt_lua_state, 1, LUA_TNUMBER);
00056          luaL_checktype(pt_lua_state, 2, LUA_TNUMBER);
00057          /* Get reference to actuator */
00058          CCI_RangeAndBearingActuator* pcAct = CLuaUtility::GetDeviceInstance<CCI_RangeAndBearingActuator>(pt_lua_state, "range_and_bearing");
00059          /* Check parameter values */
00060          size_t unIdx = lua_tonumber(pt_lua_state, 1);
00061          UInt8 unData = lua_tonumber(pt_lua_state, 2);
00062          if(unIdx < 1 || unIdx > pcAct->GetSize()) {
00063             return luaL_error(pt_lua_state, "passed index %d out of bounds [1,%d]", unIdx, pcAct->GetSize());
00064          }
00065          /* Perform action */
00066          pcAct->SetData(unIdx-1, unData);
00067          return 0;
00068       }
00069       else {
00070          return luaL_error(pt_lua_state, "robot.range_and_bearing.set_data() expects either one or two arguments");
00071       }
00072    }
00073 #endif
00074 
00075 #ifdef ARGOS_WITH_LUA
00076    /*
00077     * The stack must have no values
00078     */
00079    int LuaRABClearData(lua_State* pt_lua_state) {
00080       /* Check parameters */
00081       if(lua_gettop(pt_lua_state) != 0) {
00082          return luaL_error(pt_lua_state, "robot.range_and_bearing.clear_data() expects no arguments");
00083       }
00084       /* Perform action */
00085       CLuaUtility::GetDeviceInstance<CCI_RangeAndBearingActuator>(pt_lua_state, "range_and_bearing")->ClearData();
00086       return 0;
00087    }
00088 #endif
00089 
00090    /****************************************/
00091    /****************************************/
00092 
00093    size_t CCI_RangeAndBearingActuator::GetSize() const {
00094      return m_cData.Size();
00095    }
00096   
00097    /****************************************/
00098    /****************************************/
00099 
00100    void CCI_RangeAndBearingActuator::SetData(size_t un_idx,
00101                                             UInt8 un_value) {
00102      m_cData[un_idx] = un_value;
00103    }
00104   
00105    /****************************************/
00106    /****************************************/
00107 
00108    void CCI_RangeAndBearingActuator::ClearData() {
00109      m_cData.Zero();
00110    }
00111 
00112    /****************************************/
00113    /****************************************/
00114 
00115 #ifdef ARGOS_WITH_LUA
00116    void CCI_RangeAndBearingActuator::CreateLuaState(lua_State* pt_lua_state) {
00117       CLuaUtility::StartTable(pt_lua_state, "range_and_bearing");
00118       CLuaUtility::AddToTable(pt_lua_state, "_instance", this);
00119       CLuaUtility::AddToTable(pt_lua_state, "set_data", &LuaRABSetData);
00120       CLuaUtility::AddToTable(pt_lua_state, "clear_data", &LuaRABClearData);
00121       CLuaUtility::EndTable(pt_lua_state);
00122    }
00123 #endif
00124 
00125    /****************************************/
00126    /****************************************/
00127 
00128    void CCI_RangeAndBearingActuator::SetData(const CByteArray& c_data) {
00129       if(m_cData.Size() == c_data.Size()) {
00130          m_cData = c_data;
00131       }
00132       else {
00133          THROW_ARGOSEXCEPTION("CCI_RangeAndBearingActuator::SetData() : data size does not match, expected " << m_cData.Size() << ", got " << c_data.Size());
00134       }
00135    }
00136 
00137    /****************************************/
00138    /****************************************/
00139 
00140 }
```

---

Generated on 10 Jul 2018 for ARGoS by 
 1.6.1
